# Ultrasound-assessed lung aeration correlates with respiratory system compliance in adults and neonates with acute hypoxemic restrictive respiratory failure: an observational prospective study

**DOI:** 10.1186/s12931-022-02294-1

**Published:** 2022-12-18

**Authors:** Daniele Guerino Biasucci, Barbara Loi, Roberta Centorrino, Roberto Raschetti, Marco Piastra, Luca Pisapia, Ludovica Maria Consalvo, Anselmo Caricato, Domenico Luca Grieco, Giorgio Conti, Massimo Antonelli, Daniele De Luca

**Affiliations:** 1 Department of Emergency, Anesthesiology and Intensive Care Medicine, Fondazione Policlinico Universitario “A. Gemelli” IRCCS, Rome, Italy; 2 Division of Pediatrics and Neonatal Critical Care, A. Béclère Medical Center, Paris-Saclay University Hospitals, Public Assistance – Paris Hospitals, Paris, France; 3 Anesthesiology and Intensive Care, Fondazione IRCCS Ca’ Granda Ospedale Maggiore Policlinico, Milan, Italy; 4 Catholic University of the Sacred Heart in Rome, Milan, Italy; 5 Physiopathology and Therapeutic Innovation Unit, Paris-Saclay University, Paris, France

**Keywords:** Acute respiratory distress syndrome, Lung mechanics, Lung ultrasound, Neonates

## Abstract

**Background:**

Lung ultrasound allows lung aeration to be assessed through dedicated lung ultrasound scores (LUS). Despite LUS have been validated using several techniques, scanty data exist about the relationships between LUS and compliance of the respiratory system (Crs) in restrictive respiratory failure. Aim of this study was to investigate the relationship between LUS and Crs in neonates and adults affected by acute hypoxemic restrictive respiratory failure, as well as the effect of patients’ age on this relationship.

**Methods:**

Observational, cross-sectional, international, patho-physiology, bi-center study recruiting invasively ventilated, adults and neonates with acute respiratory distress syndrome (ARDS), neonatal ARDS (NARDS) or respiratory distress syndrome (RDS) due to primary surfactant deficiency. Subjects without lung disease (NLD) and ventilated for extra-pulmonary conditions were recruited as controls. LUS, Crs and resistances (Rrs) of the respiratory system were measured within 1 h from each other.

**Results:**

Forty adults and fifty-six neonates were recruited. LUS was higher in ARDS, NARDS and RDS and lower in control subjects (overall *p* < 0.001), while Crs was lower in ARDS, NARDS and RDS and higher in control subjects (overall *p* < 0.001), without differences between adults and neonates. LUS and Crs were correlated in adults [*r* = − 0.86 (95% CI − 0.93; − 0.76), *p* < 0.001] and neonates [*r* = − 0.76 (95% CI − 0.85; − 0.62), *p* < 0.001]. Correlations remained significant among subgroups with different causes of respiratory failure; LUS and Rrs were not correlated. Multivariate analyses confirmed the association between LUS and Crs both in adults [B = − 2.8 (95% CI − 4.9; − 0.6), *p* = 0.012] and neonates [B = − 0.045 (95% CI − 0.07; − 0.02), *p* = 0.001].

**Conclusions:**

Lung aeration and compliance of the respiratory system are significantly and inversely correlated irrespective of patients’ age. A restrictive respiratory failure has the same ultrasound appearance and mechanical characteristics in adults and neonates.

**Supplementary Information:**

The online version contains supplementary material available at 10.1186/s12931-022-02294-1.

## Background

Lung ultrasound allows a semi-quantitative assessment and monitoring of lung aeration in patients affected by acute hypoxemic respiratory failure [1, 2]. In fact, progressive changes in lung aeration are associated to specific ultrasound patterns, so that several scoring systems have been proposed to assess loss of aeration by scanning anterior, lateral, and posterior lung regions [1, 2].

Ultrasound-based semi-quantitative evaluation of lung volume has been proven to strongly correlate with extravascular lung water, oxygenation and aeration estimated with CT-scan density or computerized grey scale analysis in critically ill adults and neonates [3–8]. These data, coupled with those obtained in animal models, support the use of a lung ultrasound score (LUS) in critical care to reliably assess lung aeration at the bedside [8].

To the best of our knowledge, in patients with acute restrictive respiratory failure, scanty data exist about the relationships between LUS and lung compliance. A typical example of this condition is acute respiratory distress syndrome (ARDS), whose clinical definitions are available for patients of any age [9]. ARDS morbidity and mortality are lower in children and neonates than in adults, although they are still relevant [10–12]. Thus, it would be important to know if the age influences lung compliance and its relationship with LUS, as this can further characterize the respiratory failure and help understanding the outcome difference in patients of varying age. However, these data are lacking and we only know that, in adult ARDS patients, lung compliance correlates with the amount of aerated lung tissue being proportional to the residual ventilable lung volume: the smaller the open lung, the lower the compliance [13]. Furthermore, a recent case series reported a negative correlation between LUS and dynamic compliance in 10 adults undergoing veno-venous extra-corporeal membrane oxygenation (ECMO) [14].

There is a need for cross-disciplinary awareness, as concepts well clarified in adult or neonatal critical care are rarely verified in the alternate specialty and this may delay a comprehensive understanding of lung pathophysiology and ultrasound findings. We designed this study to investigate the relationship between LUS and the compliance of the respiratory system (Crs) in adults and neonates affected by acute hypoxemic restrictive respiratory failure, as well as the effect of patients’ age on this relationship. We hypothesized that LUS would be significantly correlated with Crs irrespective of patients’ age.

## Methods

### Design

This was an observational, cross-sectional, international, pathophysiological, bi-center study conducted between December 2019 and July 2021 in two academic referral intensive care (one neonatal and one adult) units (ICU). Two institutional review boards independently approved the study (n.9596/17/1513 and n.16/58) for adults and neonates, respectively. Informed consent was obtained from parents or guardians upon ICU admission of neonates. For ICU-admitted adults, informed consent was obtained as per local regulations, following the local Ethical Committee recommendations. The participation to the study did not change the routine clinical care. The study was conducted in accordance with the Helsinki declaration, was completely anonymous and respected local and European privacy regulations. The manuscript was prepared following STROBE guidelines [15].

### Patients

Cases consisted of adult and newborn patients admitted to ICUs for restrictive respiratory failure. To be enrolled adults must have fulfilled all the following criteria: (1) age > 18 years; (2) diagnosis of ARDS according to the Berlin definition [12]; (3) need of invasive mechanical ventilation. Neonates could have been enrolled if they fulfil all the following criteria: (1) postnatal age ≤ 7 days; (2) diagnosis of neonatal ARDS (NARDS), or diagnosis of respiratory distress syndrome (RDS; i.e., hyaline membrane disease due to primary surfactant deficiency), both according to the criteria detailed in the Montreux definition [16]; (3) need of invasive mechanical ventilation. Both RDS and NARDS patients were enrolled, since these ideally represent examples of moderate and severe restrictive respiratory failure, respectively [16, 17].

Additionally, two other groups of subjects were enrolled as controls and consisted of adult and newborn patients admitted to the ICU with no lung disease (NLD). These patients fulfilled all the following criteria: (1) need of invasive ventilation for non-pulmonary reason; (2) no need for supplemental oxygen (i.e., ventilation in room air) as well as steadily normal blood gases and vital parameters; (3) normal chest clinical examination; (4) absence of thoracic trauma and any respiratory disorder in the last month or week, in adults and neonates, respectively.

Exclusion criteria for adults with and without ARDS were: acute or chronic obstructive conditions (i.e., chronic obstructive pulmonary disease, asthma), neuromuscular diseases, interstitial lung diseases, rib cage anomalies, home long-term oxygen therapy, need for ECMO. Exclusion criteria for neonates with and without NARDS or RDS were: major congenital malformations, genetic syndromes and chromosomopathies, neuromuscular diseases, congenital lung or rib cage anomalies, airway obstruction due to meconium plugging, need for rescue high frequency oscillatory ventilation, as previously described [18], or ECMO.

### Lung mechanics measurements

Adult patients were on apneic sedation or paralyzed when clinically indicated, according to current best practice principles [19]. Volume-controlled ventilation was provided with a tidal volume (Tv) of 6 mL/kg of predicted body weight; inspiratory time and  flow were 60 L/min and 0.3 s, respectively. Respiratory rate was titrated to obtain pH between 7.35 and 7.45 with a maximal rate of 30 bpm. Positive end-inspiratory pressure (PEEP) was titrated according to current international guidelines [20] and inspired oxygen fraction (FiO_2_) was set to obtain a peripheral oxygen saturation (SpO_2_) no lower than 90%. Crs was measured in a “quasi-static” way using a standard 500 ms end-inspiratory pause to obtain plateau pressure (Pplat) and do the following calculation:

Crs = Tv/(Pplat − (PEEP + auto-PEEP)). Crs in adults was indexed to the ideal body weight. Respiratory system resistances (Rrs) were estimated as Rrs = (Peak inspiratory pressure − Pplat)/flow.

Neonates were sedated and time-cycled, pressure-regulated, assisted-controlled ventilation was provided as previously described [18]; no muscle relaxants were used. The same blood gas values of adult patients were targeted, but FiO_2_ was as low as possible to guarantee pre-ductal SpO_2_ between 90 and 95%. They were on continuous flow neonatal ventilators equipped with a low dead-space, hot-wire anemometers coupled with pressure sensors at the Y-piece to maximize the accuracy of lung mechanics assessment [21]. Sensors underwent serial technical quality controls [22] and were calibrated before each use, following manufacturers’ recommendations. Lung mechanics measurements were performed as previously described [23]: Crs and Rrs were dynamically estimated by breath-to-breath analysis shown by the ventilator software but measurements were considered only after airway suctioning, once neonates were stable and using our previously described technique to reduce ventilatory drive and increase measurement precision [24]. In detail, spontaneous breathing was temporally avoided by increasing the mechanical rate, whereas the flow was decreased to 5 L/min to reach a “quasi-static” situation. Under these conditions, when leaks were < 5% and pressure/flow-volume loops were steady, Crs and Rrs were averaged on 10 mechanical breaths. In neonates, Crs was indexed to the birth weight and was measured before surfactant administration, if any. Lung mechanics was assessed in all adults and neonates upon ICU admission as per our clinical routine and all patients were supine during the assessment.

### Lung ultrasound

Lung ultrasound was performed upon ICU admission as per our routine clinical protocols and anyway within 1 h from the lung mechanics measurements. Lung ultrasound exams were conducted with convex [5 MHz, using Xario-200® (Toshiba, Tokyo, Japan)] or micro-linear, “hockey-stick” (15 MHz, using CX-50®, Philips, Eindhoven, Netherlands) probes in adults and neonates, respectively. In both adults and neonates, ultrasound setting was as follows: gain was automatically adjusted with the dedicated software function, depth and focus were adjusted according to patients’ size and the sign of interest and no harmonics was used. Ultrasound exams were always performed by attending physicians with at least 2 years of training with daily use of lung ultrasound, or by residents in training under the supervision of the former [25]. Lung ultrasound is the first-line lung imaging technique in both recruiting ICUs, which are known to use lung ultrasound on a daily basis integrated in the clinical routine.

LUS was calculated using a standardized simple protocol already validated for both adults and neonates [5, 26]. In detail, a total of six lung areas (3 for each lung) were examined, with both transverse and longitudinal scans, while patients were supine; a score from 0 to 3 was assigned to each lung area and LUS could range from 0 to 18 (the higher the score, the worse the lung aeration). The scoring system was based on classical lung ultrasound semiology: *0* indicated a normal lung with presence of lung sliding, visible A-lines with less than three B-lines per intercostal space; *1* was given to mild alveolo-interstitial pattern, depicted by at least three B-lines or presence of multiple subpleural consolidations (with a maximal size ≤ 1 cm); *2* was attributed to a severe alveolo-interstitial pattern, represented by multiple, crowded and coalescent B-lines (i.e., “white” lung) and/or multiple subpleural consolidations separated by thickened or irregular pleura; *3* was given to severe loss of lung aeration represented by consolidations (i.e., subpleural echo-poor or tissue-like echotexture zones with size > 1 cm and irregular borders, which may also have bronchogram as mixed hypo- and hyperechogenic zones).

### Statistics

A formal sample size calculation was unfeasible since only one case series of 10 adult patients with respiratory failure reported the relationship between ultrasound-assessed lung aeration and Crs, without investigating the effect of age [14]. Thus, we decided to enroll a larger population and we choose a convenience sample size of at least 40 adult and 40 newborn patients.

Continuous variables were expressed as means (standard deviation). Categorical variables were presented as number (%). LUS and Crs were compared between adults and neonates with different respiratory conditions using one way-ANOVA followed by post hoc Sidak test, if appropriate. LUS and Crs were studied with correlation analysis using Pearson correlation coefficient (*r*) and its 95% confidence interval (CI), estimated with Fisher method. Results were also graphically shown using scatter plots analyzed with local (smoother) regression with 95% Epanechnikov kernel. The relationship between Crs and LUS was finally investigated with multivariable models built by multivariate linear regressions with backward-stepwise method. Covariates inserted in the models were: BMI, patient age (or gestational age for neonates) and the respiratory condition (considered as NLD = 0, RDS = 1, NARDS = 2 for neonates and NLD = 0, ARDS = 1 for adults). These covariates were chosen as they are known to potentially influence the severity of respiratory failure [27–31]. Multi-collinearity was evaluated considering the variance inflation factor, as previously described [32]. Birth weight was not considered as it is known to have significant multi-collinearity with the gestational age. Analyses were performed with SPSS 28 and *p* < 0.05 was considered statistically significant.

## Results

Basic details of enrolled patients are presented in Additional file 1: Table S1. In adults ARDS was triggered by COVID-19 pneumonia (n = 10), multiple trauma (n = 9) or pneumonia of other etiology (n = 4). The adults with NLD enrolled as controls were ventilated for neurosurgical reasons (n = 9) or traumatic brain injury (n = 8). In neonates, NARDS was triggered by sepsis (n = 5), pneumonia (n = 4), lung hemorrhage (n = 1), meconium aspiration (n = 1), necrotizing enterocolitis (n = 1). The neonates with NLD enrolled as controls were ventilated for hypoxic-ischemic encephalopathy due to perinatal asphyxia (n = 22) or hemodynamic instability due to diabetic hypertrophic cardiomyopathy (n = 1). Neonates were enrolled at a mean postnatal age of 1.5 (0.9) days. All patients survived and were discharged from the ICU.

**Fig. 1:**
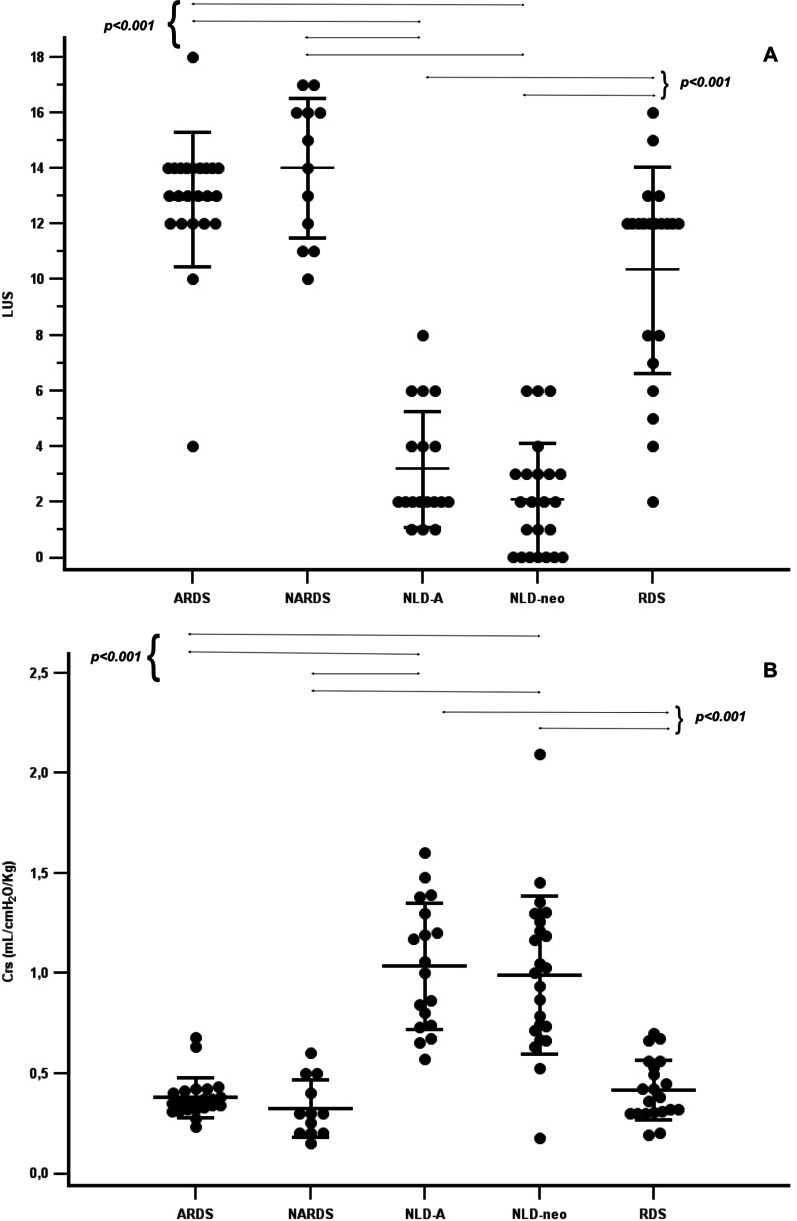
Lung ultrasound score (**A**) and compliance of the respiratory system (**B**) in all patients. Data were shown per each group of cases (adults with ARDS: N = 23; neonates with NARDS: N = 12; neonates with RDS: N = 21) and controls subjects (adults with no lung disease: N = 17; neonates with no lung disease: N = 23). Data from all patients are depicted. LUS is a dimensionless number; compliance is indexed per predicted ideal body weight and birth weight in adults and neonates, respectively. Short horizontal lines and T-bars represent means and standard deviations, respectively. Long horizontal lines represent one-to-one post hoc significant comparisons. *ARDS* acute respiratory distress syndrome, *Crs* compliance of the respiratory system, *LUS* lung ultrasound score, *NARDS* neonatal acute respiratory distress syndrome, *NLD-A* adults with no lung disease, *NLD-neo* neonates with no lung disease, *RDS* respiratory distress syndrome due to primary surfactant deficiency (i.e., hyaline membrane disease)

The Fig. 1 shows LUS and Crs in adults and neonates classified according to their respiratory condition. The distribution of mean LUS (higher in ARDS, NARDS and RDS patients, lower in control subjects with NLD) and Crs (lower in ARDS, NARDS and RDS patients, higher in control subjects with NLD) are similar in adults and neonates. Mean LUS and Crs are significantly different between patients with different respiratory conditions (overall *p* < 0.001 for both): patients with any type of restrictive respiratory failure have higher LUS and lower Crs compared to control subjects with NLD (results of post hoc significant comparisons are shown in Fig. 1).

**Fig. 2:**
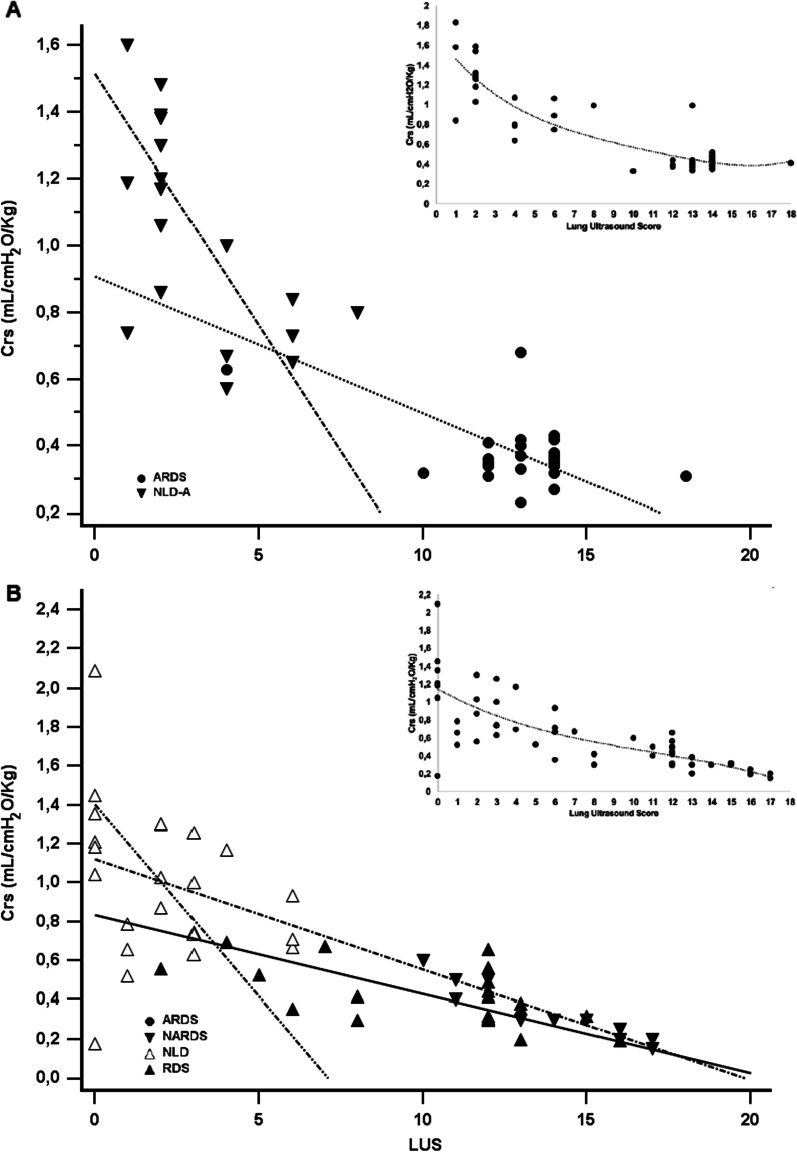
Relationship between respiratory system compliance and ultrasound-assessed lung aeration. **A** and **B** represent adults (N = 40) and neonates (N = 56), respectively. Different symbols indicate the patient subgroups. Data from subgroups and all patients (ie: cases and control subjects together) are depicted in the main figures and in the inserts, respectively. Lung ultrasound score is a dimensionless number; compliance is indexed per predicted ideal body weight and birth weight in adults and neonates, respectively. Correlation lines are shown per each subgroup (main figures), while the hatched curves are drawn for the whole adult and newborn population (inserts) and represent the best fitting data curves generated with local smoother regression. *ARDS* acute respiratory distress syndrome, *Crs* compliance of the respiratory system, *LUS* lung ultrasound score, *NARDS* neonatal acute respiratory distress syndrome, *NLD-A* adults with no lung disease, *NLD-neo* neonates with no lung disease, *RDS* respiratory distress syndrome due to primary surfactant deficiency (i.e., hyaline membrane disease)

LUS and Crs are significantly and similarly correlated in adults [*r* = − 0.86 (95% CI − 0.93; − 0.76), *p* < 0.001] and in neonates [*r* = − 0.76 (95% CI − 0.85; − 0.62), *p* < 0.001] and their relationship is shown in Fig. 2; there is no clear overlap between subgroups except, partially, between RDS and NARDS. Subgroup analysis per respiratory condition show that LUS and Crs are also significantly correlated in each subgroup of patients (NLD (adults and neonates): *r* = − 0.39 (95% CI − 0.62; − 0.09), *p* = 0.041; ARDS: *r* = − 0.46 (95% CI − 0.73; − 0.06), *p* = 0.027; NARDS: *r* = − 0.94 (95% CI − 0.98; − 0.79), *p* < 0.001; RDS: *r* = − 0.58 (95% CI − 0.81; − 0.19), *p* = 0.006. The correlation between LUS and Crs in adults remains similar if Crs is indexed per actual body weight [r = − 0.92 (95% CI − 0.95; − 0.85), p < 0.001]. There is no significant correlation between LUS and Rrs, neither in adults (*r* = 0.10, *p* = 0.952), nor in neonates (*r* = 0.113, *p* = 0.510).

Multivariate analyses confirm (Additional file 2: Table S2) that LUS is significantly and inversely associated to Crs both in adults and neonates, while patients’ age and the other considered possible confounders are not.

## Discussion

Our findings show that LUS and Crs have a mirror-like trend as they are respectively higher and lower in patients with any type of restrictive respiratory failure, than in control subjects without lung disease. Thus, LUS and Crs are significantly and inversely correlated in patients with acute hypoxemic restrictive respiratory failure, irrespective of patients’ age. These results are novel, since, to the best of our knowledge, no studies had investigated the relationship between ultrasound-assessed lung aeration and compliance, in patients of very different age, affected by acute hypoxemic restrictive respiratory failure. These findings are particularly interesting to build cross-disciplinary awareness and they may be useful for neonatal and pediatric practice, where lung ultrasound is still relatively uncommon, as they indicate how the experience in adults may help understanding patients’ pathophysiology.

Our data are also important for the following reasons. First, in patients with respiratory failure due to restrictive pathophysiology, LUS and Crs are significantly correlated and this allows to “visualize” the loss of compliance by estimating the loss of lung aeration, suggesting a possible usefulness of LUS to titrate the respiratory support [2, 33, 34]. Second, these results have been obtained in a well-selected population without any obstructive or mixed respiratory failure. Interestingly, both patients and animal models of broncho-pulmonary dysplasia (which has a mixed pattern characterized by non-homogeneously decreased compliance and increased resistances) show no correlation between LUS and Crs [35]. This is due to the small airway obstruction, as significantly obstructed lung areas are less ventilated and do not contribute to the Crs calculation [35], thus LUS may be less informative in non-restrictive disorders. Third, and most importantly, patients’ age has no significant effect on the relationship between LUS and Crs: in fact, these variables have the same tendency in adults and neonates with or without restrictive respiratory failure (Fig. 1). This is also fully consistent with the accuracy of LUS in identifying neonates affected by RDS who fail continuous positive airway pressure (CPAP) and need surfactant replacement [5, 36, 37]. Interestingly, RDS is a purely restrictive respiratory failure due to the primary surfactant deficiency and is usually milder than ARDS which has a more complex pathobiology [16]; however, CPAP failure identifies severe cases and, in fact, RDS patients enrolled in our study had LUS and Crs comparable to ARDS patients, which explains the partial overlap in Fig. 2. Finally, as shown by the multivariate analyses, the association between LUS and Crs is not influenced by patient or gestational age, nor by other covariates: this strengthens the results confirming that LUS can detect the loss of lung aeration due to a restrictive process in patients of any age.

As lung ultrasound is becoming more widely used, these findings might also be important to refine ARDS definitions. They are still mainly based on conventional radiology although modifications including lung ultrasound are available or accepted, if clinicians have sufficient expertise [38, 39]. Conversely, as ARDS has different morbidity and mortality in neonates and adults [10–12], whereas LUS and Crs follow the same trend in both types of patients, these variables are not useful to understand the reasons behind the different severity of ARDS across patients’ age. One can conclude that a restrictive respiratory failure has the same ultrasound appearance and mechanical characteristics irrespective of patients’ age, while other biological factors are responsible for the different outcomes of ARDS in neonatal and pediatric patients [40]. Conversely, LUS can generally serve as “densitometer” to assess the fluid/gas ratio, monitor its evolution and integrate clinical decision-making algorithms in patients of any age affected by respiratory failure due to a restrictive disorder [2, 41].

Our study limitations include a relatively small sample size, not free from few outliers, although it was larger than previously reported case series [14] and was represented by a homogeneous population with restrictive pathophysiology, whose main characteristics was the difference in patients’ age. The inclusion of control groups without lung disease also allowed to study more in detail the relationship between LUS and Crs. We did not recruit children beyond neonatal age, however neonatal and pediatric ARDS are known to have very similar epidemiology and pathophysiology [10], thus it is unlikely that patients of intermediate age would present different results. We used different ultrasound machines and probes for patients of different age and this was needed because of their size (convex and linear probes are always used in routine clinical care for adults and infants, respectively). Nonetheless, the ultrasound setting was identical for adults and neonates and inter-rater agreement for LUS calculation is known to be very high even when using different probes [25]. Ultrasonographers were not blinded to patients diagnosis and our data cannot directly support the use of LUS to guide alveolar recruitment and titrate respiratory support, as this requires dedicated blinded clinical trials. This was out of our scopes as our study aimed to clarify relationship between lung pathophysiology and ultrasound imaging and the effect of patient age on that relationship. We referred to the Crs, rather than lung compliance, since the partitioning is unfeasible in many neonates due to the patients’ size and, to ensure the uniformity of measurements, Crs was assessed in a “quasi-static” way in both types of patients. Finally, we did not use other techniques to measure lung volume and mechanics, such as CT-scan or gas dilution, since these are unavailable in neonates, and we wanted to keep a feasible comparison between patients of different age.

## Conclusions

In conclusion, ultrasound-assessed lung aeration appears similar in adults and neonates, with or without acute hypoxemic restrictive respiratory failure: thus, a restrictive respiratory failure has the same ultrasound appearance and mechanical characteristics irrespective of patients’ age. Consistently, lung aeration and compliance of the respiratory system are significantly and inversely correlated irrespective of patients’ age. These data improve our understanding of lung pathophysiology and ultrasound imaging.

## Supplementary Information

**Additional file 1: Table S1.** Basic population details. Data are expressed as number (%), mean (standard deviation). CRIB-II, SNAPPE-II, SAPS and LUS are dimensionless scores; compliance is indexed per predicted ideal body weight and birth weight in adults and neonates, respectively.

**Additional file 2: Table S2.** Multivariate linear regression on the relationship between respiratory system compliance and ultrasound-assessed lung aeration.

## Data Availability

DDL and DGB take responsibility for the content of the manuscript, including the data and analysis. The datasets used and/or analysed during the current study are available from the corresponding author on reasonable request.

## Abbreviations

ANOVA: Analysis of variance
ARDS: Acute respiratory distress syndrome
CI: Confidence interval
CPAP: Continuous positive airway pressure
CRIB-II: Critical risk index for babies-II
Crs: Compliance of the respiratory system
ECMO: Extra-corporeal membrane oxygenation
FiO_2_: Inspired oxygen fraction
ICU: Intensive care unit
LUS: Lung ultrasound score
NARDS: Neonatal ARDS
NLD: No lung disease
PEEP: Positive end-inspiratory pressure
Pplat: Plateau pressure
RDS: Respiratory distress syndrome
Rrs: Respiratory system resistances
SAPS-II: Simplified acute physiology score-II
SGA: Small for gestational age
SNAPPE-II: Score for neonatal acute physiology-perinatal extension-II
SpO_2_: Peripheral oxygen hemoglobin saturation
Tv: Tidal volume
