# Supplementary material for: Ultrasound-assessed lung aeration correlates with respiratory system compliance in adults and neonates with acute hypoxemic restrictive respiratory failure: an observational prospective study
Source: Respir Res. 2022 Dec 18;23:360. doi: 10.1186/s12931-022-02294-1 (PMC9759805; doi:10.1186/s12931-022-02294-1)
Supplement: Supplementary file 1 — Additional file 1: Table S1. Basic population details. Data are expressed as number (%), mean (standard deviation). CRIB-II, SNAPPE-II, SAPS and LUS are dimensionless scores; compliance is indexed per predicted ideal body weight and birth weight in adults and neonates, respectively. [file 12931_2022_2294_MOESM1_ESM.docx]

**Table S1. Basic population details.** Data are expressed as number (%), mean (standard deviation). CRIB-II, SNAPPE-II, SAPS and LUS are dimensionless scores; compliance is indexed per predicted ideal body weight and birth weight in adults and neonates, respectively. **Abbreviations**: ARDS: acute respiratory distress syndrome; Crs: compliance of the respiratory system; CRIB-II: critical risk index for babies-II; LUS: lung ultrasound score; NARDS: neonatal acute respiratory distress syndrome; NLD: no lung disease; RDS: respiratory distress syndrome due to primary surfactant deficiency (i.e.: hyaline membrane disease); Rrs: resistances of the respiratory system; SAPS-II: simplified acute physiology score-II; SGA: small for gestational age; SNAPPE-II: score for neonatal acute physiology-perinatal extension-II.

| **Adults (N=40)** | | **Neonates (N=56)** | |
| --- | --- | --- | --- |
| Age (years) | 61.9 (14.9) | Gestational age (weeks) | 33.6 (5.5) |
| Weight (Kg) | 75.4 (12) | Birth weight (g) | 2295 (1200) |
| BMI (Kg/m^2^) | 25.3 (3.3) | SGA neonates | 1 (1.8%) |
| Male sex | 26 (65%) | Male sex | 30 (53.6%) |
| SAPS-II | 36.9 (19.6) | CRIB-II / SNAPPE-II | 8.1 (3.4) / 38.5 (20.3) |
| Crs (mL/cmH_2_O/Kg) | 0.44 (0.14) | Crs (mL/cmH_2_O/Kg) | 0.63 (0.4) |
| Rrs (cmH_2_O/L/sec) | 19 (2.9) | Rrs (cmH_2_O/L/sec) | 133 (100) |
| LUS | 8.5 (5.3) | LUS | 7.7 (5.7) |
| Respiratory conditions  ARDS | 23 (57.5%) | Respiratory conditions  NARDS | 12 (21.4%) |
| NLD | 17 (42.5%) | RDS | 21 (37.5%) |
|  | | NLD | 23 (41.1%) |
