# Supplementary material for: Ultrasound-assessed lung aeration correlates with respiratory system compliance in adults and neonates with acute hypoxemic restrictive respiratory failure: an observational prospective study
Source: Respir Res. 2022 Dec 18;23:360. doi: 10.1186/s12931-022-02294-1 (PMC9759805; doi:10.1186/s12931-022-02294-1)
Supplement: Supplementary file 2 — Additional file 2: Table S2. Multivariate linear regression on the relationship between respiratory system compliance and ultrasound-assessed lung aeration. [file 12931_2022_2294_MOESM2_ESM.docx]

**Table S2. Multivariate linear regression on the relationship between respiratory system compliance and ultrasound-assessed lung aeration.** Abbreviations: BMI: body mass index; CI: confidence interval; LUS: lung ultrasound score.

|  | **B (95%CI)** | ***p*** | **Adj-R^2^** |
| --- | --- | --- | --- |
| **Adults** | | | |
| LUS | -2.8 (-4.9; -0.6) | 0.012 | 0.85 |
| BMI | -0.9 (-2.2; 0.3) | 0.143 |  |
| Age | -0.04 (-0.28; 0.20) | 0.736 |  |
| Respiratory condition | -17 (-39; 3.6) | 0.1 |  |
| **Neonates** | | | |
| LUS | -0.045 (-0.07; -0.02) | 0.001 | 0.57 |
| Gestational age | 0.001 (-0.015; 0.17) | 0.882 |  |
| Respiratory condition | 0.09 (-0.27; 0.09) | 0.330 |  |
